# A mixed-methods SWOT analysis of community pharmacy services in Saudi Arabia: stakeholder perspectives and strategic framework

**DOI:** 10.3389/fmed.2026.1728571

**Published:** 2026-02-16

**Authors:** Sultan M. Alshahrani

**Affiliations:** Department of Clinical Pharmacy, College of Pharmacy, King Khalid University, Abha, Saudi Arabia

**Keywords:** community pharmacy, health policy, healthcare reform, mixed-methods research, Saudi Arabia, stakeholder perspectives, SWOT analysis

## Abstract

**Background:**

Community pharmacies in Saudi Arabia play a significant role in healthcare access, although clinical integration remains constrained by workforce imbalance, infrastructure limitations, and regulatory fragmentation. As the healthcare system under Saudi Vision 2030 advances, emphasizing digitalization, privatization, and decentralized care, strategic evaluation of community pharmacy contributions is crucial. This study incorporates stakeholder perspectives and contemporary evidence using a mixed-methods SWOT analysis to build an evidence-based framework driving national policy and practice reform. By blending stakeholder insights with literature findings, it provides a context-specific assessment of strengths, weaknesses, opportunities, and threats impacting community pharmacy practice in the Kingdom.

**Methods:**

A convergent mixed-methods design was applied. The qualitative strand involved a structured narrative synthesis and thematic SWOT analysis of 17 peer-reviewed papers (2015–2023) following Braun and Clarke’s approach. The quantitative strand contained a 25-item Likert-scale survey of 91 pharmacy stakeholders, pharmacists, academics, regulators, industry professionals, and students across all major Saudi areas. Results from both strands were merged using joint displays and systematically translated into a TOWS matrix by cross-matching internal (strengths and weaknesses) and external (opportunities and threats) factors to develop a strategic framework aligned with Saudi Vision 2030.

**Results:**

The literature-based SWOT analysis highlighted significant strengths, including a well-developed educational infrastructure and a supportive policy environment. Weaknesses comprised inadequate clinical integration and an imbalanced workforce distribution across regions. Opportunities arose from the expansion of digital health, insurance reforms, and national workforce initiatives, while threats involved growing commercial pressures within community pharmacy practice and overlapping regulatory responsibilities. The stakeholder survey reflected substantial alignment with these patterns, identifying underuse of pharmacists’ clinical roles (M = 4.09, SD = 0.72) as a main weakness and the expansion of the private healthcare sector under Saudi Vision 2030 as the top opportunity (M = 4.44, SD = 0.61). Areas of divergence were also observed, particularly in the perceived urgency of digital health implementation.

**Conclusion:**

This mixed-methods SWOT–TOWS analysis moves beyond descriptive SWOT categorization by translating integrated stakeholder and literature evidence into a strategic framework for strengthening community pharmacy services in Saudi Arabia. The findings highlight both enabling conditions and persistent structural constraints, offering a system-level roadmap to support regulatory coherence, expanded clinical roles for pharmacists, and digital health integration in alignment with Saudi Vision 2030.

## Introduction

Community pharmacies are among the most accessible and trusted healthcare touchpoints worldwide, serving as the first point of contact for many patients (1). In recent years, their role has expanded significantly, especially during and after the COVID-19 pandemic, as pharmacists have provided essential public health services, continuity of care, and vaccination support (1). Their responsibilities have expanded beyond dispensing to encompass preventive care, medication review, chronic disease management, immunization, and health education (2, 3). This change is particularly important as healthcare systems are evolving, with increasing demands for better access, quality, and efficiency (4).

In Saudi Arabia, community pharmacies are uniquely positioned to support national health reforms under Vision 2030, which emphasizes healthcare decentralization, private-sector empowerment, and digital transformation. However, realizing this potential remains constrained by regulatory ambiguity, limited integration into the broader healthcare system, and imbalanced workforce distribution across regions (5, 6). Recent national evidence indicates that while the number of community pharmacies and licensed pharmacists has grown substantially in the last decade, service distribution and quality indicators remain uneven, particularly in rural areas (5).

Some of these shortcomings have been addressed recently by the Saudi Food and Drug Authority (SFDA) and the Saudi Ministry of Health (MOH). The policy’s intent to transform community pharmacy services is indicated by initiatives like the Wasfaty e-prescription platform, electronic health record integration, and the expansion of the pharmacist’s role in managing chronic diseases. However, persistent barriers such as outdated reimbursement models, limited clinical scope, and fragmented collaboration among providers continue to slow progress (7, 8).

Assessments of the workforce have shown improved Saudization ratios, a rise in female participation, and an increase in the number of pharmacy graduates. Nonetheless, there are still issues with insurance literacy, service equity across regions, and pharmacist training for clinical roles (9, 10). Global frameworks that have been suggested as guidelines for coordinating professional education with service readiness include the World Health Organization’s Health System Building Blocks and the International Pharmaceutical Federation’s (FIP) Development Goals (11, 12).

Healthcare organizations are increasingly using strategic planning tools like TOWS matrices and SWOT (Strengths, Weaknesses, Opportunities, and Threats) to assess system readiness and develop context-driven solutions. Although these tools have been used descriptively in previous studies, few have integrated them with empirical data from local stakeholders (13, 14). Similar patterns have been reported across other Gulf Cooperation Council and low- and middle-income countries, where pharmacists’ expanding clinical roles and health-system integration efforts mirror the transformation priorities underway in Saudi Arabia (15–17).

Our current study adopts a convergent mixed-methods design to bridge this gap. By combining a national cross-sectional survey of pharmacy stakeholders with a literature-based SWOT analysis, we integrate system-level insights with practitioner perspectives to co-develop a strategic framework that aligns with Saudi Vision 2030. This approach also reflects recent methodological advances in mixed-methods research, which emphasize the purposeful integration and contextual relevance (18). Therefore, this study integrates stakeholder perspectives with evidence from recent literature through a mixed-methods SWOT analysis to develop an evidence-based strategic framework that informs policy reform and enhances the contribution of community pharmacists to national healthcare transformation under Saudi Vision 2030.

Specifically, the objectives of this study were to: (1) identify the key strengths, weaknesses, opportunities, and threats shaping community pharmacy services in Saudi Arabia based on recent literature; (2) examine the perceptions of pharmacy and health-sector stakeholders regarding these domains; and (3) integrate both evidence sources to develop a TOWS-based strategic framework aligned with Saudi Vision 2030.

## Methods

### Study design

This study employed a convergent mixed-methods design, combining a cross-sectional stakeholder survey as the quantitative component and a literature-based SWOT analysis as the qualitative component (19). This design was deliberately chosen to allow the parallel collection and subsequent integration of stakeholder perspectives and documentary evidence, thereby enabling a comprehensive assessment of community pharmacy services at both practice and system levels. This approach was selected to address the complex nature of healthcare reform and the transformation of community pharmacy services in Saudi Arabia, allowing for the integration of empirical stakeholder evidence with documented system-level insights (6).

The survey captured the opinions and experiences of individuals working in the pharmacy profession, while the literature analysis provided an understanding of historical policy trends and professional development directions within Saudi Arabia’s evolving healthcare landscape (9). A convergent design was preferred over sequential mixed-methods alternatives because the study objective was not to build or test instruments iteratively, but rather to examine the degree of alignment, divergence, and complementarity between contemporary stakeholder perceptions and existing evidence within the same analytical timeframe. Both components were conducted concurrently, and findings were synthesized during the interpretation phase to identify convergence, divergence, and complementarity between data sources. This process followed established guidance for convergent designs, as described by Fetters et al. (20), and reporting standards outlined in the Good Reporting of Mixed Methods Studies (GRAMMS) framework by O’Cathain et al. (21).

The qualitative analysis followed the six-step thematic framework proposed by Braun and Clarke (22), ensuring methodological rigor and transparency. In addition, two global frameworks, the World Health Organization’s Health System Building Blocks (11) and the International Pharmaceutical Federation (FIP) Development Goals (23), were applied to structure the interpretation of findings and enhance policy relevance. Specifically, these frameworks were used as analytical lenses during theme mapping and validation, allowing identified SWOT elements to be examined in relation to health-system functions, workforce development, service delivery, and governance priorities.

The literature-based component represents a structured narrative synthesis rather than a full systematic review. To improve transparency and reproducibility, a systematic approach was applied for literature selection, screening, and inclusion. The process involved identifying records, removing duplicates, and performing a full-text review based on predefined eligibility criteria. This approach was intended to enhance methodological clarity without introducing procedures or reporting standards associated with formal systematic reviews. Together, these methods provided a comprehensive, triangulated understanding of the strengths, weaknesses, opportunities, and threats shaping community pharmacy practice and its alignment with Saudi Vision 2030 reforms.

## Eligibility criteria

A systematic search was conducted across PubMed, Scopus, and Google Scholar to identify studies relevant to community pharmacy practice and policy within Saudi Arabia. The search combined terms such as “community pharmacy,” “SWOT analysis,” “Saudi Arabia,” “Vision 2030,” “digital health,” and “pharmacist workforce.”

Only peer-reviewed articles published in English between 2015 and 2023 were considered. Studies were included if they (1) focused on community pharmacy services, workforce development, or policy reform in Saudi Arabia, and (2) contained empirical data or structured analyses relevant to the health-system context. Articles were excluded if they were commentaries, conference abstracts, or unrelated to pharmacy service delivery.

After screening titles, abstracts, and full texts, 17 studies met the inclusion criteria and were analyzed qualitatively. Although comprehensive database searching was undertaken, the purpose of this selection process was to support contextual synthesis rather than evidence grading or meta-analysis. This transparent, step-wise process ensured that the selected literature reflected both the historical evolution and the ongoing transformation of pharmacy practice under Saudi Vision 2030.

### Qualitative strand: SWOT analysis based on literature

Following the eligibility screening, 17 peer-reviewed studies were included for qualitative analysis. These studies focused on community pharmacy practice, workforce development, health policy, and digital transformation within the Saudi healthcare system. Each article was reviewed in full, and its key findings were carefully examined and organized under the four SWOT domains: Strengths, Weaknesses, Opportunities, and Threats to reflect the current landscape of community pharmacy services.

The analysis followed the six-step thematic process proposed by Braun and Clarke (22), ensuring a transparent and systematic organization of ideas. The primary analysis was conducted by the author, while an external subject-matter expert independently reviewed the selected studies to identify recurring patterns, and classify findings within the SWOT framework. Agreement on the final themes was reached through discussion and comparison of interpretations. Saturation was considered achieved when no new insights emerged from additional studies. The external review focused on cross-checking thematic organization and coherence, with final analytic decisions retained by the author, to increase rigor, ensure consistency, and minimize bias.

The identified themes were aligned with two global reference frameworks: the WHO Health System Building Blocks and the FIP Development Goals, enabling the analysis to link international development standards with local pharmacy practice in Saudi Arabia, thereby improving contextual relevance (11, 12). These frameworks were applied post-coding to assess thematic coverage, coherence, and policy relevance rather than as prescriptive coding templates. The final SWOT matrix formed the basis of the TOWS-based strategic framework presented in the Results section, where the literature-derived insights were translated into actionable strategies aligned with Saudi Vision 2030.

Although this strand relied solely on peer-reviewed academic literature, it is acknowledged that this approach may not capture emerging reforms and innovations documented in grey literature or policy reports.

### Quantitative strand: stakeholder survey overview

A comprehensive survey was recently conducted to gather insights from a diverse group of 91 stakeholders across six different sectors: community pharmacists, hospital pharmacists, pharmacy academics, representatives from the pharmaceutical industry, policymakers and regulators, and pharmacy students. In this study, the term “stakeholders” refers to pharmacy and health-sector professionals who directly influence, regulate, deliver, educate, or are being prepared to participate in community pharmacy services within the Saudi healthcare system. The sample size of 91 was deemed appropriate for an exploratory mixed-methods study, where the goal was to capture diverse professional perspectives rather than to achieve statistical representativeness. This size aligns with prior descriptive pharmacy workforce studies conducted in similar national contexts.

Participants were recruited using a convenience sampling approach through institutional networks, professional associations, and official communication channels. Invitations were distributed electronically via email and professional messaging groups, and participation was entirely voluntary. The survey was self-administered using an online questionnaire format. Each participant provided informed consent before accessing the survey link. To make sure a wide range of perspectives was captured, the included participants were from all five major regions of Saudi Arabia: Central, Eastern, Western, Northern, and Southern. Notably, the majority of respondents brought between 1 and 20 years of experience to the table, and the gender distribution was nearly balanced, ensuring a well-rounded perspective.

The survey was structured around the four SWOT analysis dimensions, consisting of five statements per area rated using a five-point Likert scale (1 denoting “Strongly Disagree” and 5 “Strongly Agree”). The questionnaire items were initially generated through an extensive review of literature on community pharmacy practice, workforce development, and policy reforms in Saudi Arabia. A preliminary pool of 30 items was drafted and refined to 25 after review by a panel of five subject-matter experts, including two academic pharmacists, one hospital pharmacist, one policy representative, and one industry professional. The same instrument was administered across all stakeholder categories to allow comparison of shared system-level perceptions rather than role-specific performance evaluation. These statements were specifically designed to elicit perceptions regarding accessibility, training, regulatory frameworks, integration, digitalization, and sector-specific threats. Examples of the survey items included: “Community pharmacies are widely accessible to the public,” “Integration of community pharmacies into the national healthcare system is limited,” and “Vision 2030 facilitates private-sector growth in healthcare.”

A pilot test was conducted with 10 individuals to ensure the clarity and comprehensibility of the questions before the main survey. The pilot group reflected all stakeholder categories and was asked to provide structured feedback on item clarity, logical flow, and response burden. Feedback from this exercise led to minor wording adjustments and improved alignment of items under the four SWOT domains. The expert panel also assessed the instrument’s content and construct soundness through consensus, ensuring each item accurately reflected its intended domain to confirm validity.

The reliability of the survey tool was confirmed, with Cronbach’s alpha coefficients for each SWOT area ranging from 0.814 to 0.881, indicating good internal consistency. Data analysis was performed using SPSS (version 27), focusing on descriptive statistics such as means, standard deviations, and rankings. Subgroup analyses were not conducted because the study was not powered for inferential comparisons and was designed to generate exploratory, system-level insights rather than role-specific conclusions. Although a large number of professionals and regions were represented in the survey, it is important to note that the use of convenience sampling and the modest sample size may limit the generalizability of findings. However, the data provide valuable insight about the opinions of important pharmacy stakeholders within Saudi Arabia’s evolving healthcare landscape.

### Integration strategy

Integration was carried out during the interpretation stage in line with the reasoning of the convergent mixed-methods design. Integration was primarily analytic in nature, with findings from the stakeholder survey and literature-based SWOT analysis first organized into a joint display matrix that aligned strengths, weaknesses, opportunities, and threats with corresponding stakeholder perceptions. This side-by-side comparison enabled the identification of points of convergence, divergence, and complementarity across both datasets.

Afterward, the synthesized SWOT outcomes were systematically converted into a TOWS framework, which extended the analysis from descriptive categorization to actionable strategic planning. Specifically, the internal (strengths and weaknesses) and external (opportunities and threats) elements were cross-matched to derive four strategy types: SO (leveraging strengths to seize opportunities), WO (addressing weaknesses through opportunities), ST (using strengths to mitigate threats), and WT (minimizing weaknesses and threats simultaneously). This process represents a second level of integration, where mixed-methods findings were operationalized into policy-relevant strategic options rather than interpreted in isolation.

The integration of both qualitative and quantitative findings was performed collaboratively by two researchers through iterative consensus discussions to ensure consistency and transparency in interpretation. The final synthesis adopted a narrative weaving approach, allowing for a cohesive strategic interpretation supported by triangulated data from stakeholder insights and literature evidence.

### Ethical considerations

The study protocol was reviewed and approved by the Research Ethical Committee at King Khalid University (approval no. KKU-51-2025-17). Before data collection, written informed consent was obtained from all participants with a clear explanation of the study purpose and procedures. Data were collected anonymously, and no personally identifying information was recorded. The study adhered to the ethical principles outlined in the Declaration of Helsinki (24). Since the qualitative component utilized secondary data, no patient-level data were accessed or analyzed.

### Quality assurance

The methods were carefully designed to ensure rigor and reliability across both strands of the study. For the literature synthesis, the recognized standards for qualitative analysis were applied, using multiple data sources and transparent coding procedures to strengthen the trustworthiness of the findings. In the quantitative component, the survey instrument demonstrated strong internal consistency across all SWOT domains, lending credibility to the outcomes. By combining insights from both the literature and survey findings, as well as using global health frameworks, we were able to create a more meaningful interpretation of the results that can really inform policy decisions.

Nonetheless, certain methodological limitations should be acknowledged. The stakeholder survey involved a relatively small and non-random sample of 91 participants, which may limit generalizability. Similarly, the literature synthesis relied on a focused set of peer-reviewed publications, potentially excluding relevant grey or policy sources. Additionally, patient perspectives were not included, even though they represent a critical component of healthcare evaluation. These limitations are recognized and should be considered when interpreting the study’s findings.

## Results

### Participant characteristics

A total of 91 stakeholders completed the survey, showcasing a well-balanced distribution across community pharmacists (15.4%), hospital pharmacists (14.3%), academics (18.7%), pharmaceutical industry professionals (16.5%), regulators (17.6%), and pharmacy students (17.6%). The demographic characteristics of the 91 survey participants are presented in Table 1. Participants were geographically distributed across all five administrative regions of Saudi Arabia, and they were almost evenly split by sex (50.5% female and 49.5% male). Most respondents reported having between 1 and 20 years of experience (75.8%), ensuring that insights were gathered from both early-career and seasoned professionals.

**Table 1:** Participant demographics (*N* = 91).

Variable	Category	Frequency (*n*)	Percentage (%)
Role	Community pharmacist	14	15.4%
	Hospital pharmacist	13	14.3%
	Academic	17	18.7%
	Industry	15	16.5%
	Regulator	16	17.6%
	Student	16	17.6%
Region	Central	17	18.7%
	Western	18	19.8%
	Eastern	17	18.7%
	Northern	19	20.9%
	Southern	20	22.0%
Experience	<1 year	17	18.7%
	1–5 years	17	18.7%
	6–10 years	14	15.4%
	11–20 years	24	26.4%
	>20 years	19	20.9%
Gender	Male	45	49.5%
	Female	46	50.5%

“Experience” refers to participants’ cumulative professional or training-related exposure within the pharmacy sector, categorized by total years of experience reported.

### Qualitative strand results

A total of 17 peer-reviewed studies met the inclusion criteria for the literature-based SWOT analysis. Each study was reviewed for relevance, focus, and contribution to the four SWOT domains. Their characteristics and utilization summaries are presented in Supplementary Table S1, which outlines how each study informed the identification of specific strengths, weaknesses, opportunities, and threats within community pharmacy practice in Saudi Arabia. The thematic analysis revealed recurring patterns such as workforce localization and professional competency development as key strengths; limited clinical integration and inconsistent regulatory enforcement as weaknesses; Vision 2030-driven reforms and digital health expansion as major opportunities; and fragmented interprofessional collaboration as a persistent threat. These findings collectively informed the development of the integrated SWOT–TOWS framework that guided the strategic interpretation of results.

### Reliability of SWOT domains

Table 2 summarizes the internal consistency of the SWOT-based survey domains. Each SWOT domain showed strong internal consistency. Cronbach’s alpha coefficients ranged from 0.814 to 0.881, confirming the reliability of the Likert-scale constructs. For clarity, survey findings are presented separately under each SWOT domain (Tables 3–6), while maintaining the overall integrated structure of the 25-item framework.

**Table 2:** Cronbach’s alpha values by SWOT domain.

Domain	Cronbach’s alpha	Interpretation
Strengths	0.843	Acceptable
Weaknesses	0.814	Acceptable
Opportunities	0.845	Acceptable
Threats	0.881	Acceptable

**Table 3:** Perceived strengths of community pharmacy services.

Item code	Statement	Mean (M)	Std. dev. (SD)
S1	Community pharmacies are widely accessible to the public.	4.29	0.66
S2	The presence of qualified pharmacy graduates enhances service delivery.	4.10	0.68
S3	Pharmacists are generally trusted by the public.	4.03	0.72
S4	Community pharmacies are well-positioned to participate in primary care.	3.97	0.74
S5	Most pharmacies maintain basic service standards.	3.81	0.69

**Table 4:** Perceived weaknesses of community pharmacy services.

Item code	Statement	Mean (M)	Std. dev. (SD)
W1	Limited integration into the national healthcare system.	4.09	0.72
W2	Absence of performance-based regulatory incentives.	4.01	0.77
W3	Undefined scope for clinical pharmacy services.	3.92	0.75
W4	Inconsistent continuing education and training.	3.85	0.80
W5	Inadequate communication with other healthcare providers.	3.77	0.81

**Table 5:** Perceived opportunities in community pharmacy development.

Item code	Statement	Mean (M)	Std. dev. (SD)
O1	Vision 2030 supports private-sector healthcare expansion.	4.44	0.61
O2	National digital platforms (e.g., Wasfaty) create service innovation opportunities.	4.32	0.70
O3	Increased demand for chronic disease care expands pharmacists’ roles.	4.17	0.73
O4	Insurance reforms may incentivize professional pharmacy services.	4.05	0.76
O5	Public awareness of pharmacists’ clinical role is growing.	3.98	0.75

**Table 6:** Perceived threats to community pharmacy services.

Item code	Statement	Mean (M)	Std. dev. (SD)
T1	Over-commercialization threatens professional credibility.	4.24	0.64
T2	Fragmentation between SFDA and MOH creates policy confusion.	4.13	0.70
T3	Profit-driven models limit clinical service prioritization.	4.02	0.74
T4	Lack of national service standardization.	3.94	0.72
T5	Limited monitoring of professional conduct.	3.81	0.78

### Strengths identified by stakeholders

The most highly rated strength identified was the widespread accessibility of community pharmacies, with the statement “Community pharmacies are widely accessible to the public” receiving the highest mean score (M = 4.29, SD = 0.66). Additionally, other strengths that were well-rated included the availability of qualified pharmacy graduates (M = 4.10, SD = 0.68) and the general public’s trust in community pharmacists (M = 4.03, SD = 0.72). The key strengths identified by stakeholders are outlined in Table 3.

### Weaknesses in the current system

The highest-rated weakness identified was the limited integration of community pharmacies within the national healthcare system (M = 4.09, SD = 0.72). This was closely followed by concerns regarding the absence of performance-based regulation (M = 4.01, SD = 0.77) and ambiguities surrounding clinical service mandates (M = 3.92, SD = 0.75) (see Table 4).

### Opportunities aligned with vision 2030

Opportunities perceived by stakeholders to align with Saudi Vision 2030 goals are detailed in Table 5. Stakeholders identified the most significant opportunity as aligning pharmacy sector reforms with the Vision 2030 initiatives, which received a mean score of 4.44 and a standard deviation of 0.61. The use of digital health platforms like Wasfaty, increased insurance coverage, and the rising need for pharmaceutical care in the treatment of chronic illnesses were other noteworthy opportunities.

### Threats to professional and systemic growth

Table 6 outlines the threats identified by stakeholders. The primary concern was the increasing commercialization of community pharmacies, which was seen as undermining their professional credibility (M = 4.24, SD = 0.64). Additional issues included regulatory overlap, inconsistency in service delivery, and retail-driven operational models.

### Domain-level SWOT comparison

In our comprehensive analysis across all four domains, the Opportunities category emerged as the standout area, boasting the highest overall mean score of 4.19 (SD = 0.56). This high score reflects high stakeholder ratings for reform-related factors, including digital innovation and alignment with Vision 2030 initiatives. This pattern indicates a generally positive assessment of reform potential, particularly in relation to digital innovation and alignment with Vision 2030 initiatives.

The Strengths category came in second with a mean score of 4.04 (SD = 0.63). These scores reflect high ratings for accessibility, workforce capacity, and perceived readiness for expanded service delivery.

On the other hand, the Threats category received a score of 4.03 (SD = 0.60), while the Weaknesses category received a slightly lower score of 3.93 (SD = 0.67). Despite relatively high mean scores, the Threats and Weaknesses domains highlight persistent structural and regulatory challenges. Stakeholders reported particular concern regarding limited clinical integration and increasing commercialization of community pharmacy practice.

Overall, the domain-level results indicate the coexistence of strong enabling conditions alongside identifiable systemic constraints. These domain-level results indicate the coexistence of enabling conditions alongside identifiable systemic constraints. Table 7 summarizes comparative domain-level scores across the SWOT framework. As shown, Opportunities and Strengths received the highest mean ratings, while Weaknesses and Threats were rated slightly lower.

**Table 7:** Domain-level summary of mean scores and standard deviations.

SWOT domain	Mean (M)	Std. dev. (SD)	Interpretation
Opportunities	4.19	0.56	Highest perceived domain
Strengths	4.04	0.63	High institutional assets
Threats	4.03	0.60	Systemic risks present
Weaknesses	3.93	0.67	Clear areas needing reform

### Mixed-methods integration and interpretation

**Figure 1:**
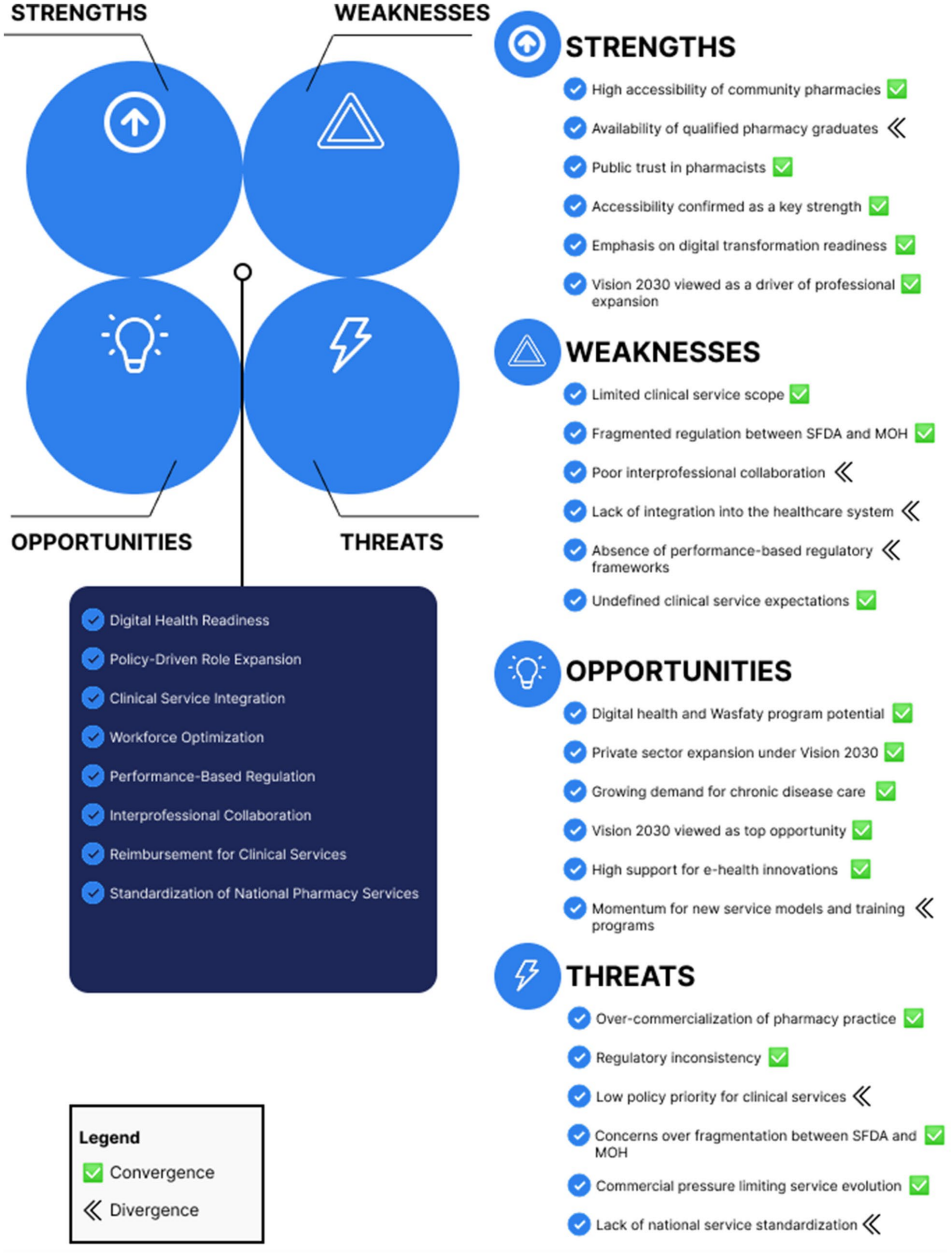
Integrated SWOT insights: comparison of literature synthesis and stakeholder survey findings. This joint display highlights areas of convergence (shared priorities) and divergence (gaps between literature and stakeholder perceptions) across the four SWOT categories. The matrix supports data triangulation and strategy formulation.

The results of the stakeholder survey were systematically incorporated using a joint display matrix approach in order to improve the validity and transparency of the SWOT analysis that was derived from the literature. The two datasets were triangulated through this integrative process, enabling identification of areas of convergence and divergence between stakeholder perspectives and the literature. Figure 1 presents this integration using a joint display matrix, aligning literature-derived SWOT themes with corresponding stakeholder survey results to visually represent areas of convergence and divergence across the four domains.

Based on a comprehensive analysis of 17 academic sources, the qualitative SWOT synthesis found several important obstacles that stand in the way of advancement in the healthcare industry. Regulatory fragmentation, persistent workforce issues, and underdeveloped clinical pathways were important obstacles. These findings showed alignment with stakeholder survey results, particularly in relation to regulatory ambiguity (T2) and limited system integration (W1).

However, the analysis also found some significant differences between stakeholder perspectives and the literature. Stakeholders expressed a pressing need for immediate action, despite the literature’s consistent presentation of digital transformation as a long-term strategic imperative. They specifically listed “Wasfaty and e-health innovation” (Opportunity 2-O2) as one of the best chances, suggesting that current policy frameworks may not keep up with the pace of digital initiatives. This divergence suggests differences in perceived urgency between stakeholders and the existing literature regarding the pace of digital health implementation.

Furthermore, both strands of analysis converged on the pivotal role of Vision 2030 as a strategic catalyst (Opportunity 1-O1) in guiding the national healthcare agenda. This shared recognition underscores the framework’s potential to provide actionable direction and mobilize resources effectively.

What is new in this study: To our knowledge, this work is one of the first efforts in the Saudi context to combine a national stakeholder survey with a targeted literature-based SWOT synthesis and to translate those complementary data into an operational TOWS strategic matrix. Unlike prior descriptive SWOT exercises in the region [e.g., Rasheed et al., (6); Alrasheedy, (5)], our mixed-methods integration highlights not only domain-level agreement but specific, actionable mismatches, for example, the stakeholder-driven urgency around Wasfaty adoption versus the literature’s characterization of digital transformation as a longer-term goal, and converts these into prioritized strategy cells in the TOWS matrix. This integrative approach, therefore, moves beyond description to produce a pragmatic, evidence-anchored set of strategic options tailored to Vision 2030 timelines and implementation realities.

The development of a TOWS-based strategic framework that highlights multiple crucial pathways is supported by the integration of these findings:

- Enhancing integration into healthcare systems through the expansion of digital services (Opportunity 2 → Weakness 1)
- Restructuring regulatory frameworks to reduce ambiguity and expedite procedures (Threat 2 → Strength 2)
- Making use of skilled graduates’ abilities to promote innovation and advancement (Strength 2 → Opportunity 3)

**Figure 2:**
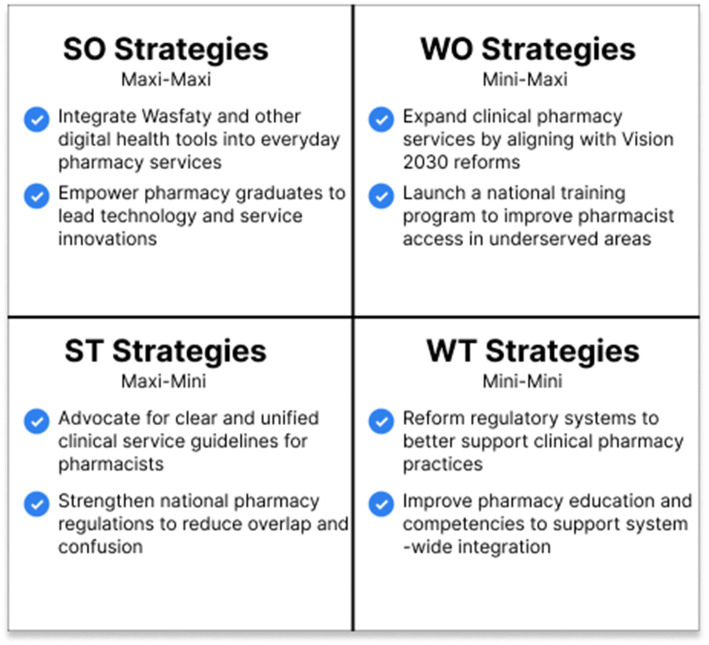
Proposed strategic framework for community pharmacy improvements: TOWS matrix. This framework synthesizes survey and literature findings into four strategic quadrants: SO (Maxi–Maxi): leverage strengths to capitalize on opportunities. WO (Mini–Maxi): overcome weaknesses using available opportunities. ST (Maxi–Mini): use internal strengths to counter threats. WT (Mini–Mini): minimize weaknesses and avoid threats through reform.

The resulting TOWS matrix is presented in Figure 2, outlining a multi-dimensional reform strategy based on combined stakeholder insights and literature synthesis.

The joint insights derived from both the literature and stakeholder inputs have been synthesized in the Discussion section of the analysis. These insights serve as a foundational component for constructing the final Strategic Framework, which will guide policy recommendations aimed at fostering a more integrated and responsive healthcare system.

## Discussion

This study integrated findings from a literature-based SWOT analysis and a nationwide stakeholder survey using a convergent mixed-methods design to examine the current status and strategic potential of community pharmacy services in Saudi Arabia (20). Following the integration principles described by Clark (25) and Fetters et al. (20), the combination of both strands provided a holistic understanding of systemic strengths, gaps, and reform opportunities. This approach enabled triangulation between stakeholder experiences and existing policy directions, thereby strengthening validity.

The highest-rated strengths identified were the accessibility of community pharmacies, an increasing number of qualified pharmacy graduates, and the public’s trust in pharmacists (26, 27). These findings are consistent with recent local studies, which show that both pharmacists and patients perceive Saudi community pharmacies as highly accessible and essential for primary healthcare delivery (26, 27). When compared internationally, similar patterns have been observed in other Gulf Cooperation Council (GCC) countries and high-income contexts. Studies from the United Arab Emirates and Qatar reported comparable public confidence and ease of access but noted a limited transition toward clinically oriented services (6, 15). Likewise, research from Oman and Bahrain emphasized pharmacists’ untapped potential in primary care delivery within community settings (15). In high-income nations such as the United States and Australia, community pharmacists are increasingly providing medication reviews, vaccinations, and public health interventions as part of integrated healthcare systems (2, 3). Taken together, these comparisons suggest that the proposed SWOT–TOWS framework is adaptable beyond Saudi Arabia, particularly in health systems pursuing expanded pharmacy roles within decentralized and digitally enabled care models.

On the other hand, we found a few areas that require improvement. Performance-based standards remain underdeveloped, and community pharmacists’ clinical roles are still evolving. Importantly, this does not indicate structural regulatory conflict between the Ministry of Health (MOH) and the Saudi Food and Drug Authority (SFDA). Rather, as clarified, the issue pertains to variation in implementation, lack of unified service protocols, and differing institutional standards. The regulatory framework itself is well established; what remains fragmented are the practice guidelines and performance indicators guiding service delivery. These systemic constraints continue to limit full integration into the national health system (28, 29).

The future holds numerous opportunities, particularly with the implementation of Saudi Vision 2030 reforms. The rise of digital health platforms, such as Wasfaty, and policies supporting pharmacists in managing chronic diseases and preventative care have been highlighted as promising developments. (30, 31). These trends also align with the World Health Organization’s Health System Building Blocks and the International Pharmaceutical Federation (FIP) Development Goals, which emphasize health workforce transformation, digital integration, and patient-centered practice (28, 32).

We must also be aware of significant challenges that could hinder progress. In this study, the term “over-commercialization” is defined as the predominance of retail- and profit-driven business models that prioritize product sales over clinical and patient-centered pharmacy services. Such an approach risks constraining the evolution of patient-centered care within community pharmacies (26, 33). Similarly, the perceived inconsistencies between MOH and SFDA activities reflect differing administrative scopes rather than legal contradictions. The main challenge lies in the absence of unified national practice standards and service protocols that ensure consistency across sectors (29, 34).

A key contribution of this study lies in its explicit mixed-methods integration. Methodologically, it demonstrates the value of combining stakeholder-generated empirical data with a structured narrative synthesis to move beyond descriptive SWOT analyses. Scholarly, it contributes context-specific evidence on community pharmacy transformation within a rapidly reforming health system. From a policy perspective, it translates integrated findings into a TOWS-based strategic framework that directly aligns with national reform priorities under Saudi Vision 2030.

A further strength of this analysis lies in identifying where stakeholder perceptions diverged from the published literature. For example, while published studies highlighted limited public trust in some Middle Eastern contexts, Saudi stakeholders reported very high trust in pharmacists’ competence and accessibility. Conversely, participants expressed greater concern about workforce training gaps and digital readiness than most prior studies, suggesting that professional expectations are shifting faster than academic reporting. Additionally, stakeholders were more optimistic about private-sector readiness to expand clinical roles than what was reflected in existing research, revealing a forward-looking optimism about the sector’s potential.

We found that there was a broad consensus throughout the integration process on topics such as the need for policy reform, the confusion caused by inconsistent regulations, and the preparedness of digital health. However, the emphasis on new service models, like home care, structured medication reviews, and vaccination delivery, varied across respondents and the literature. This divergence demonstrates the evolving expectations of pharmacists and emphasizes the importance of continuous professional development and policy alignment. By explicitly contrasting stakeholder and literature perspectives, the study underscores both progress made and opportunities still to be seized in transforming community pharmacy practice in Saudi Arabia (26, 33).

### Implications for policy, practice, and education

These results underscore the need for a comprehensive, legally supported overhaul of Saudi Arabia’s community pharmacy services (31). Strategic directions in line with national priorities under Saudi Vision 2030 are presented by the proposed TOWS matrix. Current practice gaps can be filled, especially by activating new service areas through the use of existing strengths such as accessibility and a qualified workforce (28).

Reforms that integrate community pharmacists into the national care continuum should be taken into consideration by policymakers, especially in the areas of primary care, chronic illness management, and digital health systems. To standardize service procedures and clarify pharmacists’ clinical and collaborative roles across settings, regulatory agencies such as the SFDA and MOH must continue to coordinate their efforts through unified practice standards rather than through fragmented operational guidelines (29, 34).

Educational institutions and professional associations play an essential role in shaping the future of healthcare. It’s important to update our curricula to include topics like clinical readiness, digital health skills, and teamwork across different professions. By doing this, we can align our efforts with the FIP Development Goals and WHO frameworks for universal health coverage and integrated care that puts people at the center (11, 12, 26, 27, 32).

Beyond identifying challenges and opportunities, the integrated TOWS matrix developed in this study serves as an actionable policy roadmap. It offers structured guidance for aligning regulatory modernization, digital transformation, and workforce capacity-building with the Saudi Vision 2030 health reform agenda. The framework also supports evidence-informed policy formulation by prioritizing strategies that strengthen clinical service delivery, optimize human resources, and enhance public trust in the pharmacy sector. In this regard, the Vision 2030-driven reform aligns with broader national development models that emphasize social transformation through community engagement and professional empowerment, as reflected in recent Saudi reform initiatives across other sectors (35).

### Strengths of the mixed methods approach

In order to produce findings that are not only more dependable but also tailored to the particular context, we integrated stakeholder feedback with insights from the literature in this study. For instance, stakeholders emphasized digital health initiatives as top priorities ready for implementation, whereas prior studies largely highlighted their broader significance. The stakeholder survey also underscored the necessity of financial incentives and clear regulatory frameworks, aspects that had only been mentioned in passing in earlier literature. These findings also align with previous national initiatives emphasizing the need for strategic workforce planning and sustained pharmaceutical care development in Saudi Arabia (36).

To improve interpretability and practical application, we employed joint displays (see Figure 1) and a strategic framework (see Figure 2). This approach exemplifies the advantages of mixed methods in addressing complex health service challenges, as outlined by Fetters et al. (20). Moreover, Creswell and Clark (37) argue that convergent designs provide a comprehensive understanding by leveraging the complementary strengths of qualitative and quantitative data, while Tashakkori and Teddlie (38) highlight their ability to generate findings that are both broad and deep, thereby enhancing policy relevance.

### Limitations and future research

There are several limitations to be aware of. First, despite the comprehensiveness of the literature synthesis, only 17 studies were included, which might have left out pertinent unpublished data or grey literature. This narrow evidence base limits the breadth of contextual insights available and may therefore restrict the generalizability of the qualitative findings to all aspects of the rapidly evolving Saudi healthcare system. Second, the relatively small size (*n* = 91) of the stakeholder sample may restrict generalizability, particularly for regional sub-analyses, even though it covered a wide range of roles. Hence, the perspectives captured should be interpreted as indicative rather than representative of the entire pharmacy sector. In addition, the aggregation of diverse stakeholder groups within a single analytical framework may mask role-specific differences in priorities or experiences; however, this approach was intentional and appropriate for an exploratory, system-level study aimed at identifying shared structural and strategic issues. Accordingly, subgroup analyses were not conducted, as the study was not designed or powered for inferential, role-specific comparisons. Third, causal interpretations and long-term evaluations of reforms are not possible due to the cross-sectional survey design.

Together, these limitations suggest that while the study provides meaningful strategic insights, its conclusions should be viewed as exploratory and context-specific rather than universally generalizable. Importantly, patient perspectives were not directly captured in this study, despite the central emphasis on patient-centered care within Saudi Vision 2030. Future research should include longitudinal follow-up of Vision 2030 pharmacy reforms, regional comparisons of implementation success, and role-specific as well as patient-centered qualitative investigations to better understand how community pharmacy reforms are experienced at the point of care. Additional information about workforce planning and service delivery outcomes may be obtained through the continued use of mixed methods, such as embedded and multistage designs.

## Conclusion

The current study takes an in-depth look at community pharmacy services in Saudi Arabia, using a blend of literature-based SWOT analysis and feedback from stakeholders. By combining these approaches, we provide an integrated, system-level perspective on priority strengths, constraints, and strategic directions shaping the sector. The insights from both the research and those working in the field show that while community pharmacies excel in areas like accessibility and workforce training, there are also important challenges to address, such as variations in practice standards and the dominance of commercial models that limit clinical service delivery.

To address these issues, we developed a TOWS-based strategic framework that translates integrated evidence into policy-relevant directions for coordinating community pharmacy operations with the health objectives of Saudi Vision 2030. Key actions include aligning regulatory implementation across agencies, strengthening the integration of pharmacists into primary and digital health services, and shifting incentives toward clinical performance rather than product-based models.

While the findings provide valuable system-level insights, they should be interpreted within the limits of the study’s modest sample size and literature scope. The absence of direct patient perspectives further highlights the need for complementary future research.

In summary, we demonstrate that realizing the full potential of community pharmacies in Saudi Arabia requires coordinated policy implementation, targeted workforce development, and the practical application of the proposed TOWS framework as a roadmap for sustainable, patient-centered healthcare transformation. The framework provides a structured foundation for policymakers and healthcare leaders seeking to operationalize Saudi Vision 2030 priorities within community pharmacy practice.

## Data Availability

The original contributions presented in the study are included in the article/Supplementary material, further inquiries can be directed to the corresponding author.
