# Supplementary material for: A mixed-methods SWOT analysis of community pharmacy services in Saudi Arabia: stakeholder perspectives and strategic framework
Source: Front Med (Lausanne). 2026 Feb 16;13:1728571. doi: 10.3389/fmed.2026.1728571 (PMC12950606; doi:10.3389/fmed.2026.1728571)
Supplement: Supplementary file 1 [file Data_Sheet_1.pdf]

Supplementary Tables

Supplementary Table S1: Utilization of Key Studies in SWOT Analysis

SI. NO.	Citation	SWOT Category	Utilization Summary
1	Alhazmi et al. [26]	Weakness	Highlighted limited clinical integration in community pharmacies.
2	Bajuayfir et al. [27]	Strength/Weakness	Evaluated patient trust and identified service gaps.
3	Almaghaslah et al. [29]	Opportunity	Aligned workforce strategy with FIP competencies and Vision 2030.
4	Almaghaslah [9]	Threat	Addressed workforce disparities and regulatory inconsistencies.
5	AlRuthia et al. [4]	Weakness	Provided a demographic breakdown of pharmacists' nationalities.
6	Albekairy et al. [37]	Strength	Served as a baseline workforce capacity study.
7	DiPietro Mager & Bright [2]	Opportunity	Showed the potential public health contributions of pharmacists.
8	Al Dali & Bawazir [8]	Strength	Demonstrated pharmacists' expanding roles in public health.
9	Almalki et al. [36]	Threat	Identified systemic delivery and equity constraints.
10	Albejaidi & Nair [10]	Opportunity	Reviewed Saudization policy and workforce localization.
11	Khaliq [35]	Threat	Described health system governance issues.
12	Alomi et al. [25]	Opportunity	Projected future demand for clinical pharmacists.
13	Weihrich [13]	Methodological	Provided theoretical grounding for SWOT methodology.
14	Braun & Clarke [22]	Methodological	Informed thematic coding and analysis procedures.

15	Helms & Nixon [14]	Methodological	Validated SWOT in strategic health planning.
16	Saseen et al. [33]	Weakness	Supported case for clinical pharmacy integration.
17	Udoh et al. [28]	Opportunity	Offered cross-country comparative insight.

Supplementary Table S2: SWOT Matrix Elements Identified

**Category**	**Key Element**
Strength	Robust education infrastructure
Strength	Government policy alignment with Vision 2030
Weakness	Limited clinical service provision
Weakness	Workforce maldistribution (urban-rural)
Opportunity	E-health platforms and insurance integration
Opportunity	NCD demand and decentralization of care
Threat	Retail consolidation and regulatory instability
Threat	Role ambiguity and limited institutional support

Supplementary Table S3: Strategic TOWS Matrix for Community Pharmacy Reform

**Strategy Type**	**Strategic Recommendation**
SO	Expand digital chronic care services using workforce and policy capacity.
WO	Utilize e-health and insurance to close service and geographic gaps.
ST	Align regulatory frameworks with Vision 2030 to reduce market threats.
WT	Reform pharmacist scope and incentivize rural practice to mitigate dual constraints.

Supplementary Table S4: Strategic TWOS Matrix for Community Pharmacy in Saudi Arabia

**Strategy Type**	**Description**	**Strategic Action**

**SO Strategies**(Strengths + Opportunities)	Use strengths to capitalize on external opportunities (Vision 2030, digitalization, Saudization)	- Leverage pharmacy accessibility (S1) to roll out nationwide digital screening and vaccination services (O2, O5) - Use public trust (S3) to promote pharmacist-led chronic disease management in alignment with Vision 2030 goals (O1, O4) - Expand pharmacist counseling roles using digital platforms supported by regulatory reform (S5 + O3)
**WO Strategies**(Weaknesses + Opportunities)	Overcome weaknesses by leveraging opportunities in policy, workforce, and tech	- Launch training programs on insurance systems (W1) under Vision 2030's healthcare workforce agenda (O3) - Invest in CPD and digital tool integration to address skill and documentation gaps (W3, W4 + O2) - Incentivize pharmacy-primary care collaboration to close coordination gaps (W5 + O4)
**ST Strategies**(Strengths + Threats)	Use internal strengths to mitigate external threats (competition, fragmentation)	- Use existing patient relationships (S3) to build loyalty and differentiate from non-pharmacist retailers (T1) - Promote structured services (S4) to meet regulatory expectations and reduce fragmentation (T3, T5) - Utilize pharmacy network reach (S1) to run public awareness campaigns (T2)
**WT Strategies**(Weaknesses + Threats)	Minimize weaknesses and defend against threats	- Strengthen enforcement of pharmacy practice regulations to prevent dilution of services (W2 + T1, T3) - Embed basic insurance and e-health literacy in undergraduate pharmacy education (W1 + T5) - Align private pharmacy innovation funds with Vision 2030 investment channels to reduce economic pressure (W4 + T4)

Supplementary Table S5: Vision 2030 Alignment Highlights

Pillar	TWOS Link	Impact
**Workforce Nationalization (Saudization)**	WO, SO	CPD for insurance, clinical roles, and leadership supports localization
**Digital Health Enablement**	SO, WO	Enables pharmacist-patient interaction, data documentation, and scalability

**Private Sector Expansion**	ST, WT	Strengthens pharmacy positioning against retail threats and regulatory lag
**Preventive Health Promotion**	SO, WO	Enables pharmacies to deliver accessible, affordable screening and education

Supplementary Table S6: Stakeholder Perspectives on Community Pharmacy Services

**Question**	**Options**
1. Role/Position	☐ Community Pharmacist ☐ Hospital Pharmacist ☐ Pharmacy Academic ☐ Pharmaceutical Industry Representative ☐ Policymaker/Regulator ☐ Pharmacy Intern/Student
2. Region	☐ Central ☐ Western ☐ Eastern ☐ Northern ☐ Southern
3. Years of Experience	☐ <1 year ☐ 1–5 years ☐ 6–10 years ☐ 11–20 years ☐ >20 years
4. Gender (optional)	☐ Male ☐ Female ☐ Prefer not to say

Supplementary Table S7: SWOT Statement Ratings (5-Point Likert Scale; N = 91)

**Scale:**				
1 = Strongly Disagree	2 = Disagree	3 = Neutral	4 = Agree	5 = Strongly Agree

Supplementary Figures

Supplementary Figure S1: Distribution of Studies by SWOT Category

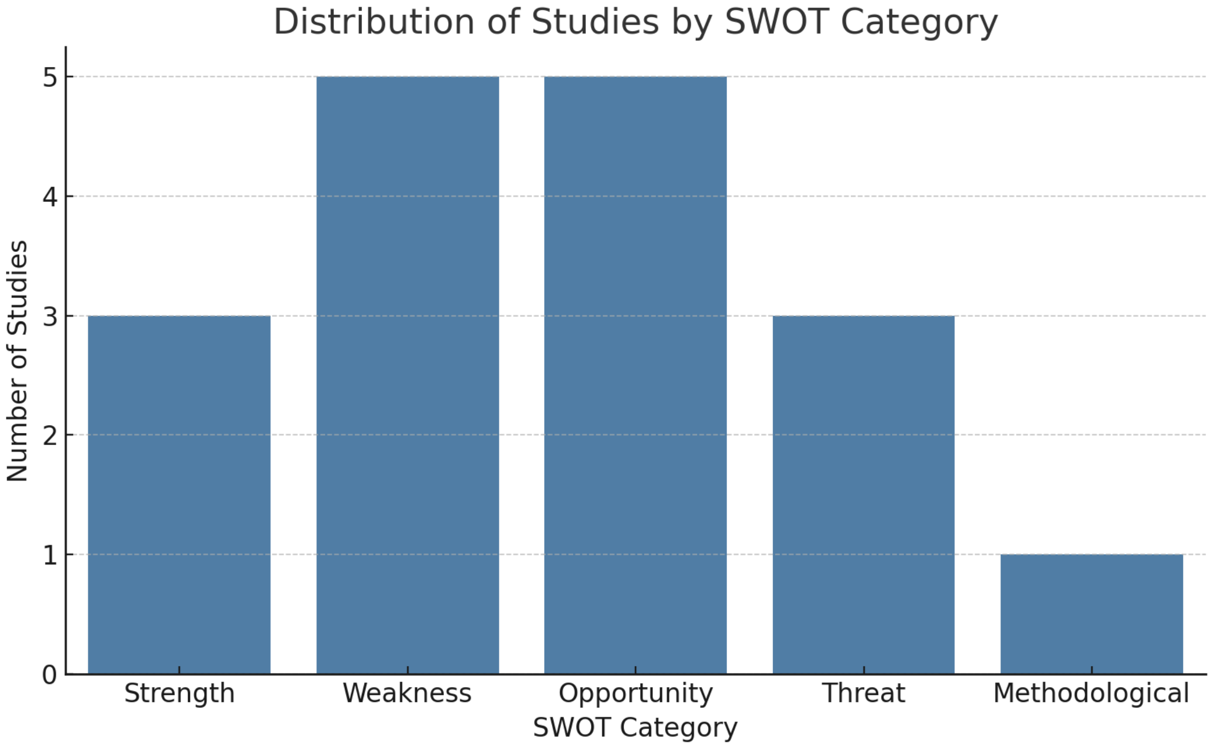

Supplementary Figure S2: Proportion of Studies Supporting SWOT Domains

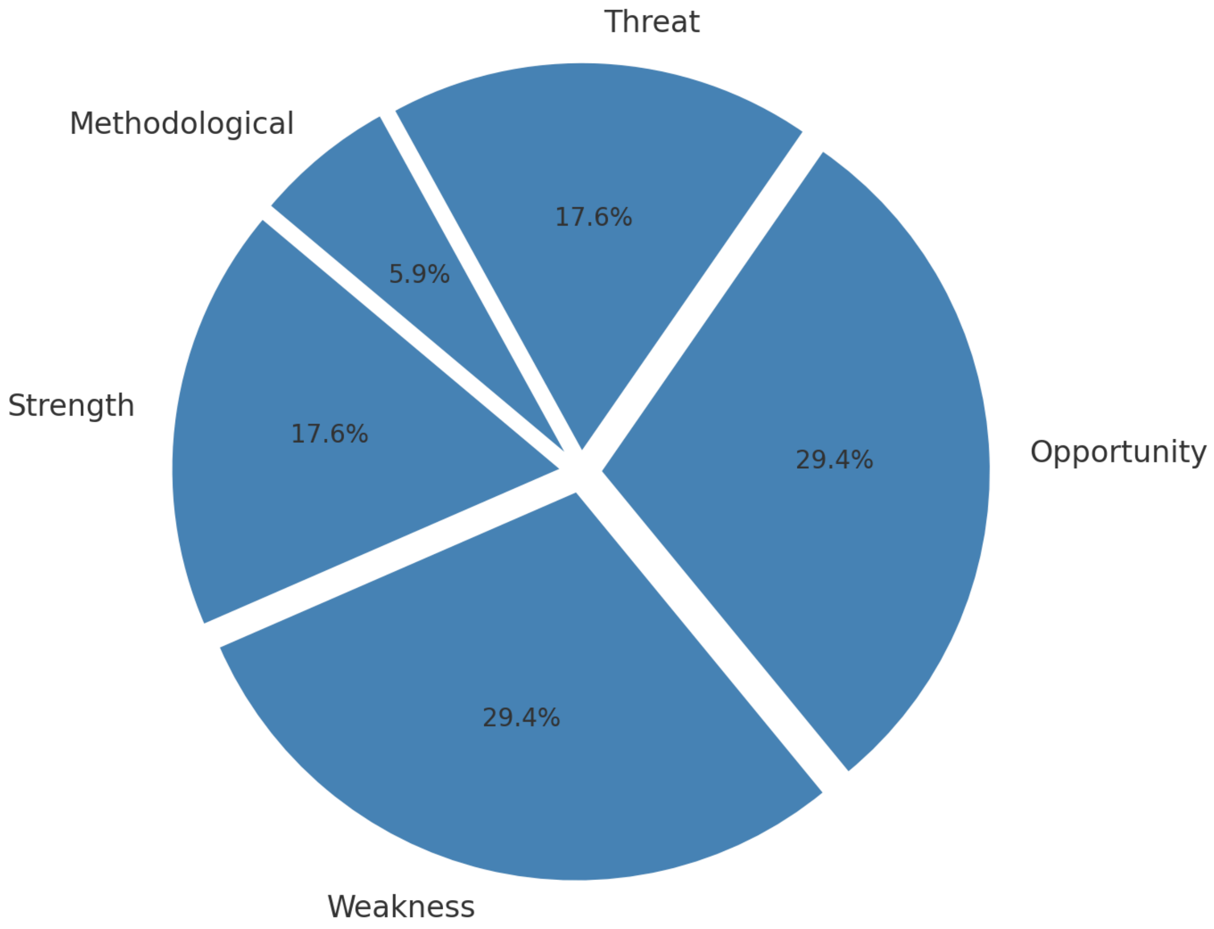
Threat
17.6%
Methodological
5.9%
Strength
17.6%
Opportunity
29.4%
Weakness
29.4%
